# Supplementary material for: RNA N6-methyladenosine modulates endothelial atherogenic responses to disturbed flow in mice
Source: eLife. 2022 Jan 10;11:e69906. doi: 10.7554/eLife.69906 (PMC8794471; doi:10.7554/eLife.69906)
Supplement: Figure 1—source data 1. [file elife-69906-fig1-data1.zip › figure 1-source data 1.pptx]

## Slide 1
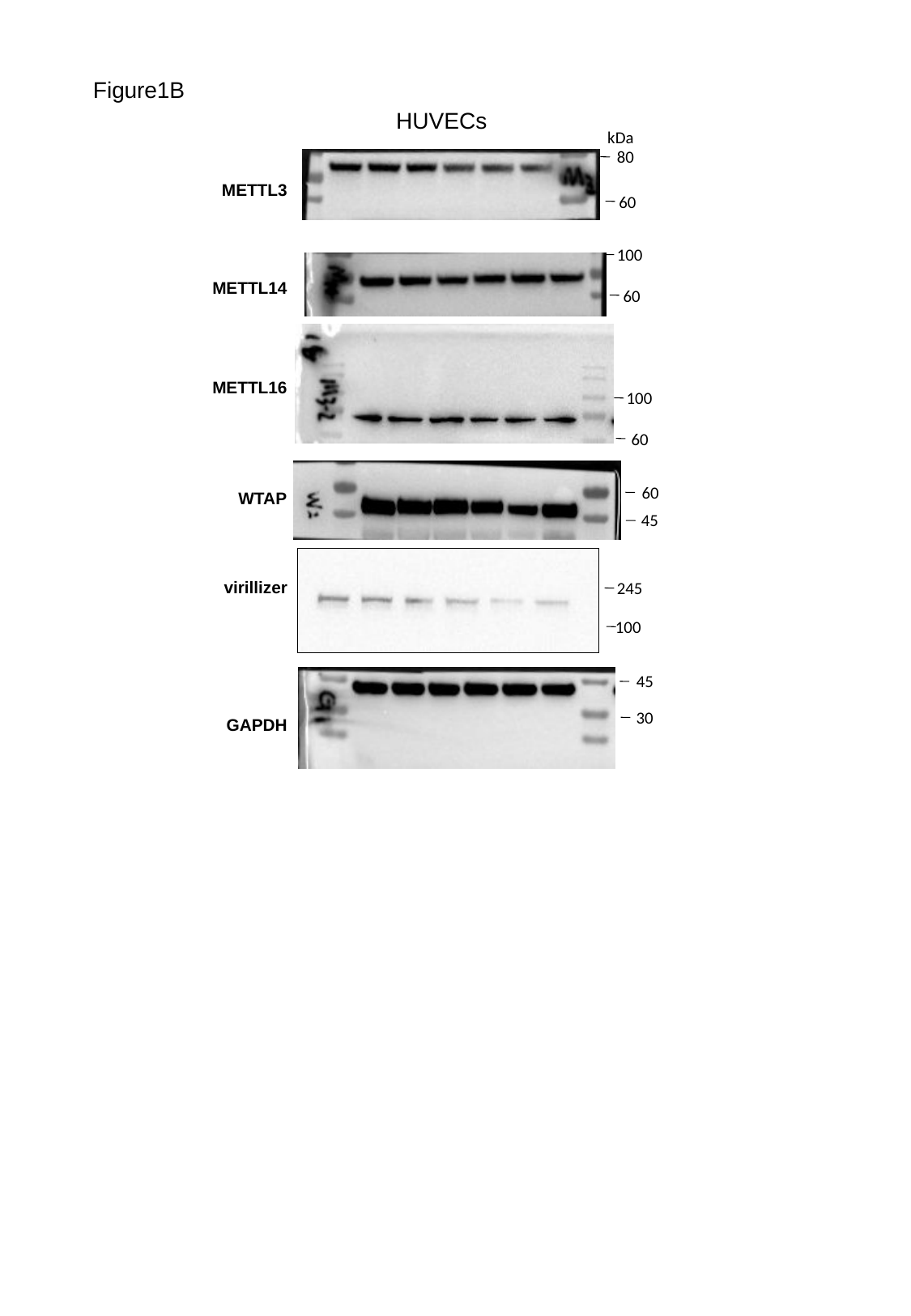

Figure1B
HUVECs
kDa
80
METTL3
60
100
METTL14
60
METTL16
100
60
WTAP
60
45
virillizer
245
100
45
GAPDH
30

## Slide 2
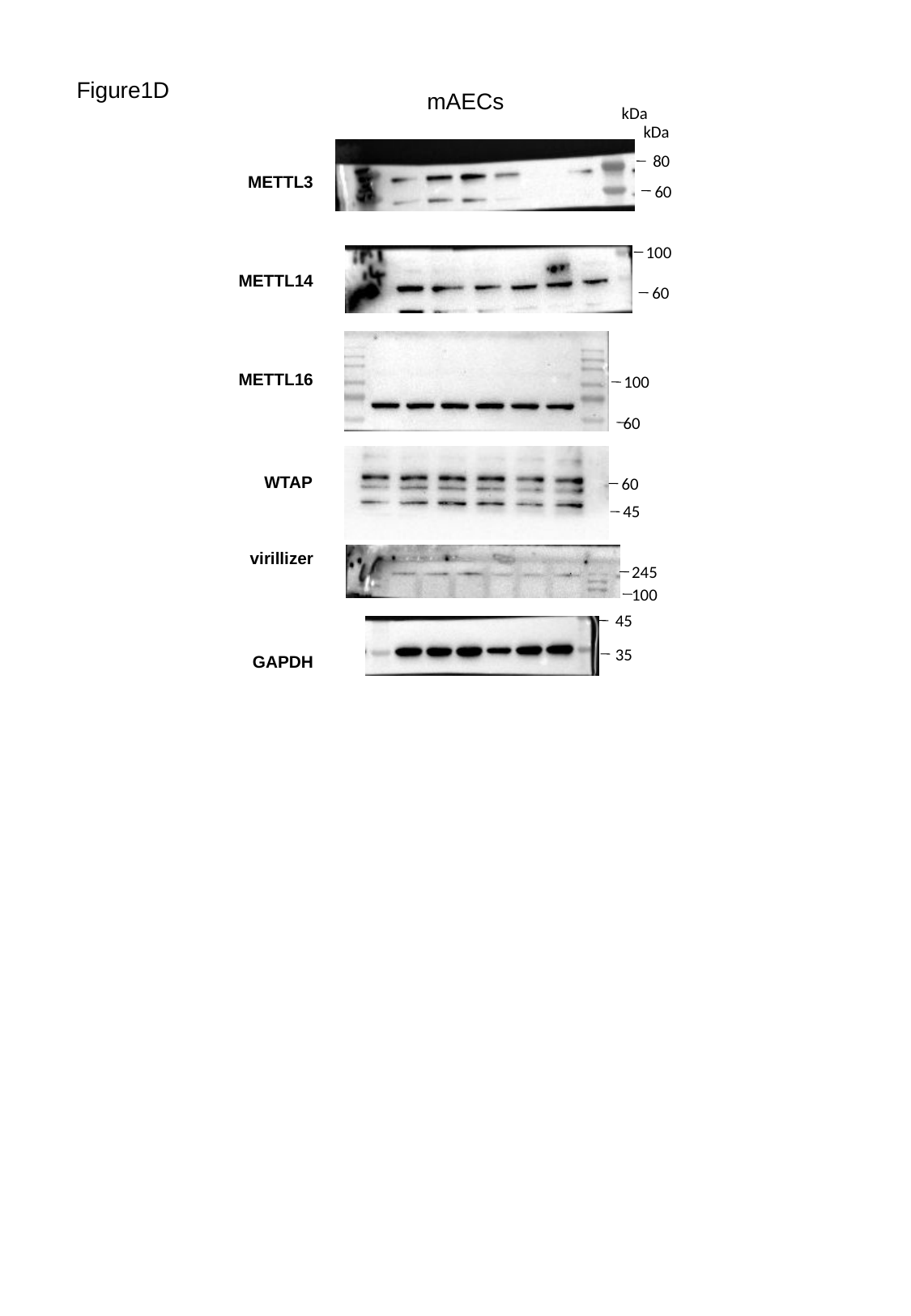

Figure1D
mAECs
kDa
kDa
80
METTL3
60
100
METTL14
60
METTL16
100
60
WTAP
60
45
virillizer
245
100
45
GAPDH
35
