# Supplementary figures and images for: RNA N6-methyladenosine modulates endothelial atherogenic responses to disturbed flow in mice
Source: eLife. 2022 Jan 10;11:e69906. doi: 10.7554/eLife.69906 (PMC8794471; doi:10.7554/eLife.69906)

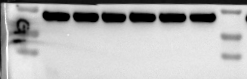

Supplement: Figure 1—source data 1. [file elife-69906-fig1-data1.zip › Figure 1-source data 1/fig 1B/GAPDH.tiff]

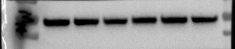

Supplement: Figure 1—source data 1. [file elife-69906-fig1-data1.zip › Figure 1-source data 1/fig 1B/METTL14.tiff]

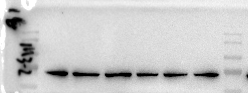

Supplement: Figure 1—source data 1. [file elife-69906-fig1-data1.zip › Figure 1-source data 1/fig 1B/METTL16.tiff]

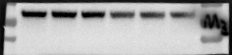

Supplement: Figure 1—source data 1. [file elife-69906-fig1-data1.zip › Figure 1-source data 1/fig 1B/METTL3.tiff]

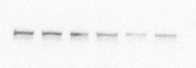

Supplement: Figure 1—source data 1. [file elife-69906-fig1-data1.zip › Figure 1-source data 1/fig 1B/virillizer.Tif]

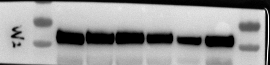

Supplement: Figure 1—source data 1. [file elife-69906-fig1-data1.zip › Figure 1-source data 1/fig 1B/WTAP.tiff]

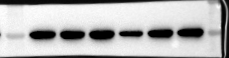

Supplement: Figure 1—source data 1. [file elife-69906-fig1-data1.zip › Figure 1-source data 1/fig 1D/GAPDH.tiff]

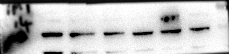

Supplement: Figure 1—source data 1. [file elife-69906-fig1-data1.zip › Figure 1-source data 1/fig 1D/METTL14.tiff]

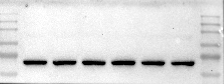

Supplement: Figure 1—source data 1. [file elife-69906-fig1-data1.zip › Figure 1-source data 1/fig 1D/METTL16.tiff]

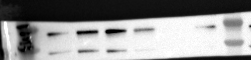

Supplement: Figure 1—source data 1. [file elife-69906-fig1-data1.zip › Figure 1-source data 1/fig 1D/METTL3.tiff]

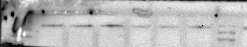

Supplement: Figure 1—source data 1. [file elife-69906-fig1-data1.zip › Figure 1-source data 1/fig 1D/virillizer.tiff]

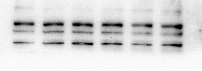

Supplement: Figure 1—source data 1. [file elife-69906-fig1-data1.zip › Figure 1-source data 1/fig 1D/WTAP.tiff]

## Slide 1
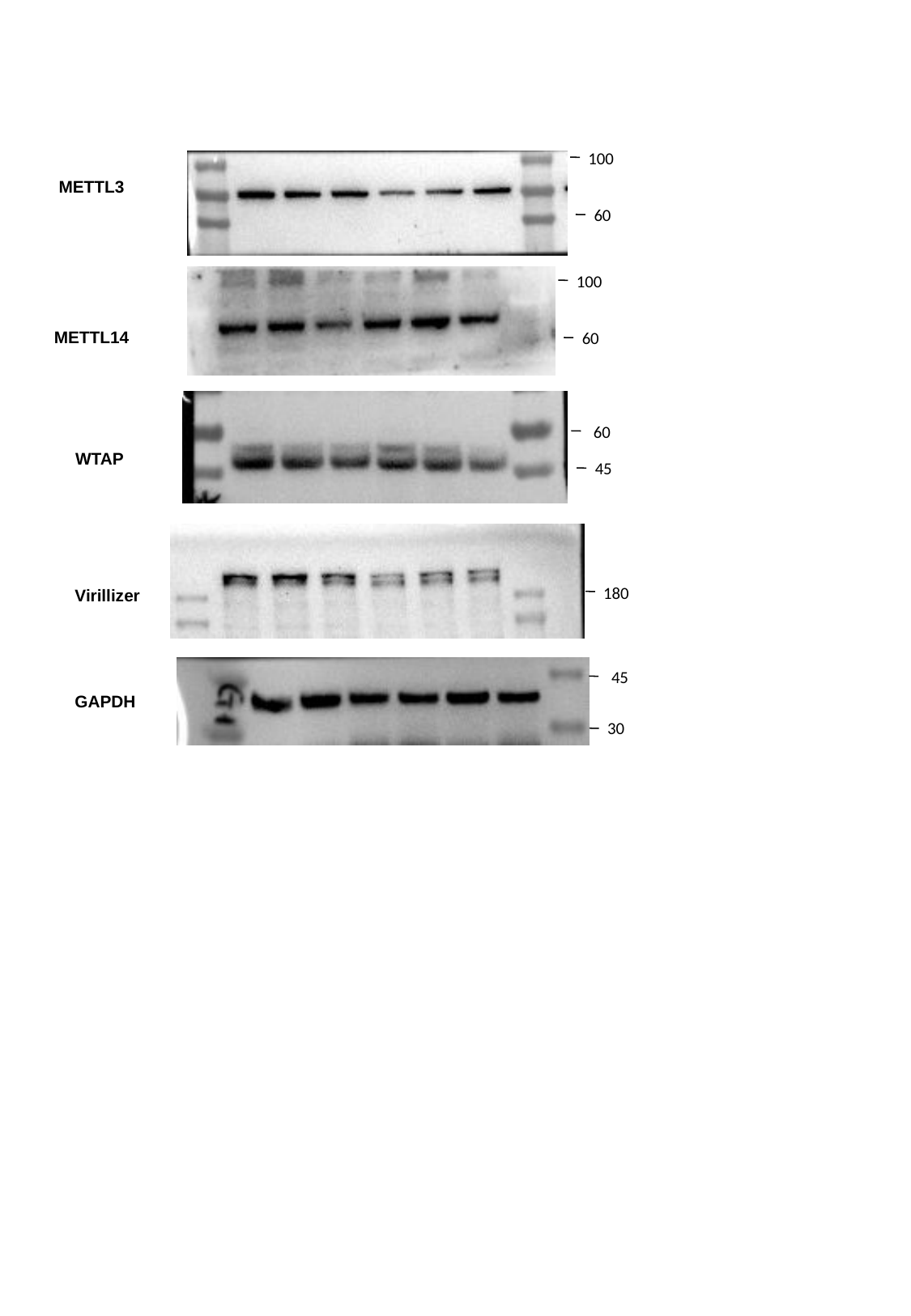

100
METTL3
60
100
METTL14
60
60
WTAP
45
Virillizer
180
45
GAPDH
30

Supplement: Figure 1—figure supplement 1—source data 1. [file elife-69906-fig1-figsupp1-data1.zip › Figure 1-figure supplement 1-source data 1.pptx]

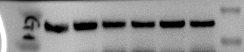

Supplement: Figure 1—figure supplement 1—source data 1. [file elife-69906-fig1-figsupp1-data1.zip › Figure 1-figure supplement 1-source data 1/GADPH.tiff]

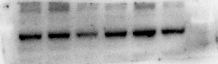

Supplement: Figure 1—figure supplement 1—source data 1. [file elife-69906-fig1-figsupp1-data1.zip › Figure 1-figure supplement 1-source data 1/METTL14.tiff]

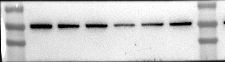

Supplement: Figure 1—figure supplement 1—source data 1. [file elife-69906-fig1-figsupp1-data1.zip › Figure 1-figure supplement 1-source data 1/METTL3.tiff]

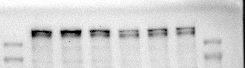

Supplement: Figure 1—figure supplement 1—source data 1. [file elife-69906-fig1-figsupp1-data1.zip › Figure 1-figure supplement 1-source data 1/virillizer.tiff]

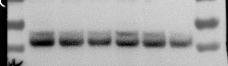

Supplement: Figure 1—figure supplement 1—source data 1. [file elife-69906-fig1-figsupp1-data1.zip › Figure 1-figure supplement 1-source data 1/WTAP.tiff]

## Slide 1
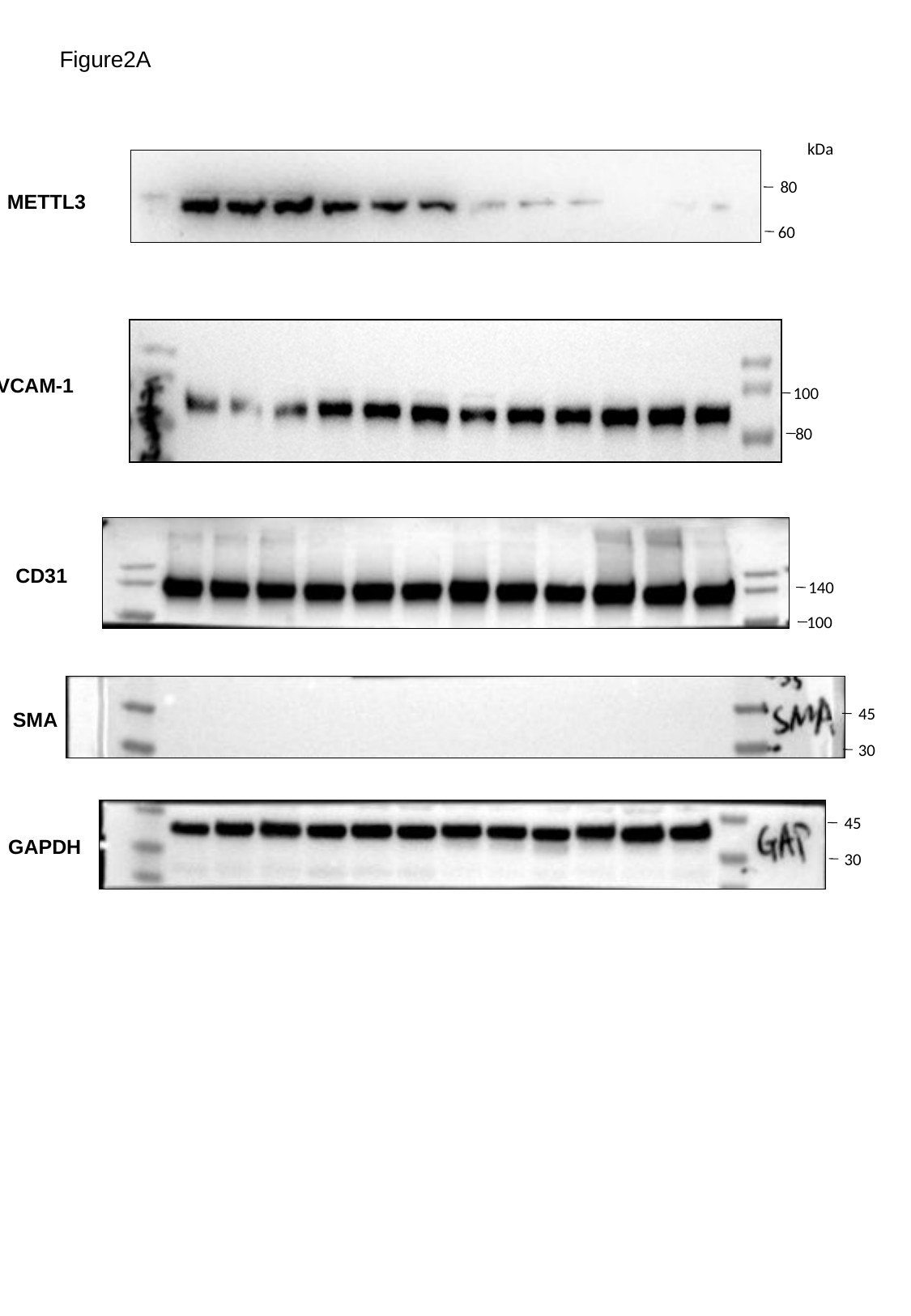

Figure2A
kDa
80
METTL3
60
VCAM-1
100
80
CD31
140
100
SMA
45
30
45
GAPDH
30

Supplement: Figure 2—source data 1. [file elife-69906-fig2-data1.zip › figure 2-source data-1.pptx]

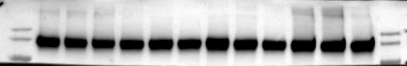

Supplement: Figure 2—source data 1. [file elife-69906-fig2-data1.zip › figure 2-source data 1/CD31.tiff]

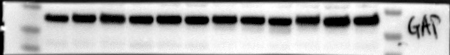

Supplement: Figure 2—source data 1. [file elife-69906-fig2-data1.zip › figure 2-source data 1/GAPDH.tiff]

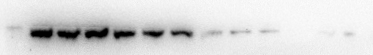

Supplement: Figure 2—source data 1. [file elife-69906-fig2-data1.zip › figure 2-source data 1/METTL3.tiff]

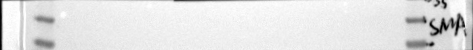

Supplement: Figure 2—source data 1. [file elife-69906-fig2-data1.zip › figure 2-source data 1/SMA.tiff]

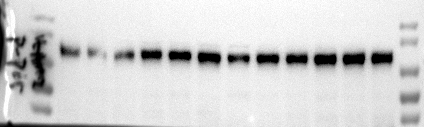

Supplement: Figure 2—source data 1. [file elife-69906-fig2-data1.zip › figure 2-source data 1/VCAM-1.tiff]

## Slide 1
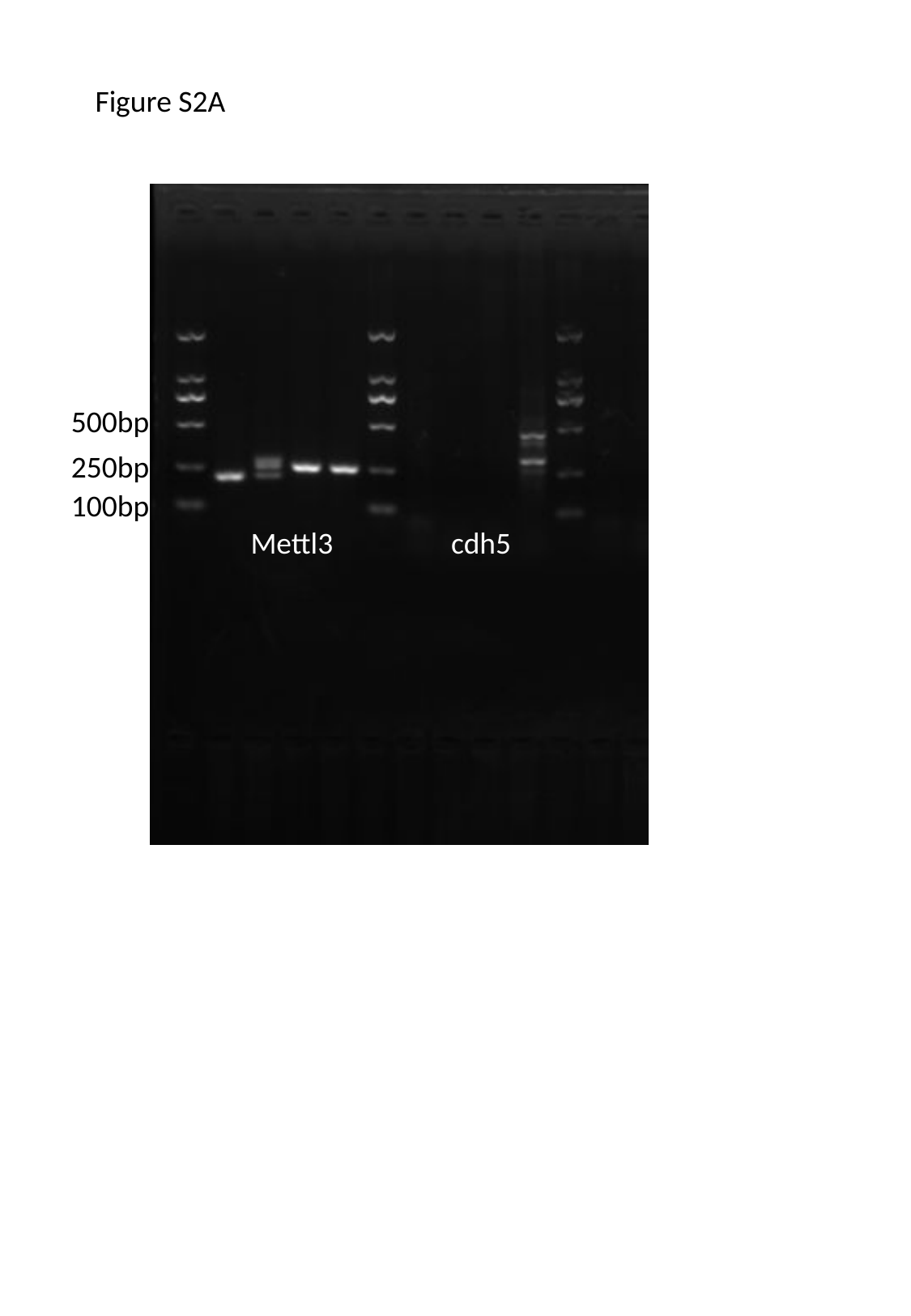

Figure S2A
500bp
250bp
100bp
Mettl3
cdh5

Supplement: Figure 2—figure supplement 1—source data 1. [file elife-69906-fig2-figsupp1-data1.zip › Figure 2-figure supplement 1-source data 1/METTL3 and Cdh5.pptx]

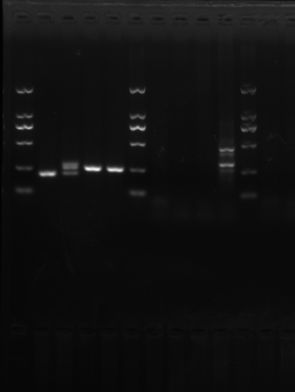

Supplement: Figure 2—figure supplement 1—source data 1. [file elife-69906-fig2-figsupp1-data1.zip › Figure 2-figure supplement 1-source data 1/METTL3 and Cdh5.tiff]

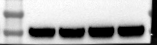

Supplement: Figure 4—source data 1. [file elife-69906-fig4-data1.zip › Figure 4-source data 1/fig4 A/AKT.tiff]

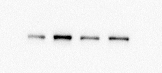

Supplement: Figure 4—source data 1. [file elife-69906-fig4-data1.zip › Figure 4-source data 1/fig4 A/EGFR.tiff]

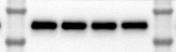

Supplement: Figure 4—source data 1. [file elife-69906-fig4-data1.zip › Figure 4-source data 1/fig4 A/ERK-6.tiff]

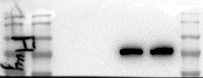

Supplement: Figure 4—source data 1. [file elife-69906-fig4-data1.zip › Figure 4-source data 1/fig4 A/flag.tiff]

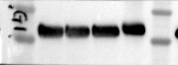

Supplement: Figure 4—source data 1. [file elife-69906-fig4-data1.zip › Figure 4-source data 1/fig4 A/GAP-1.tiff]

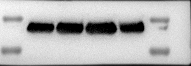

Supplement: Figure 4—source data 1. [file elife-69906-fig4-data1.zip › Figure 4-source data 1/fig4 A/GAP-2.tiff]

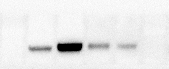

Supplement: Figure 4—source data 1. [file elife-69906-fig4-data1.zip › Figure 4-source data 1/fig4 A/P-AKT.tiff]

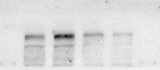

Supplement: Figure 4—source data 1. [file elife-69906-fig4-data1.zip › Figure 4-source data 1/fig4 A/P-EGFR.tiff]

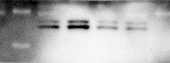

Supplement: Figure 4—source data 1. [file elife-69906-fig4-data1.zip › Figure 4-source data 1/fig4 A/p-erk-3-9_╕▒▒╛-2.tiff]

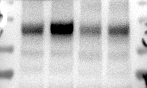

Supplement: Figure 4—source data 1. [file elife-69906-fig4-data1.zip › Figure 4-source data 1/fig4 A/VCAM-1.tiff]

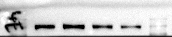

Supplement: Figure 4—source data 1. [file elife-69906-fig4-data1.zip › Figure 4-source data 1/fig4 C/EGFR.tiff]

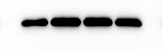

Supplement: Figure 4—source data 1. [file elife-69906-fig4-data1.zip › Figure 4-source data 1/fig4 C/GAPDH.tiff]

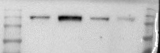

Supplement: Figure 4—source data 1. [file elife-69906-fig4-data1.zip › Figure 4-source data 1/fig4 C/p-EGFR.tiff]

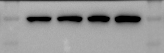

Supplement: Figure 4—source data 1. [file elife-69906-fig4-data1.zip › Figure 4-source data 1/fig4 E/AKT.tiff]

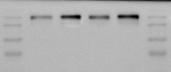

Supplement: Figure 4—source data 1. [file elife-69906-fig4-data1.zip › Figure 4-source data 1/fig4 E/EGFR.tiff]

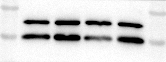

Supplement: Figure 4—source data 1. [file elife-69906-fig4-data1.zip › Figure 4-source data 1/fig4 E/ERK.tiff]

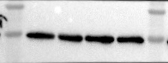

Supplement: Figure 4—source data 1. [file elife-69906-fig4-data1.zip › Figure 4-source data 1/fig4 E/GAP1.tiff]

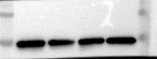

Supplement: Figure 4—source data 1. [file elife-69906-fig4-data1.zip › Figure 4-source data 1/fig4 E/GAP2.tiff]

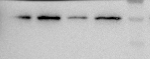

Supplement: Figure 4—source data 1. [file elife-69906-fig4-data1.zip › Figure 4-source data 1/fig4 E/P-AKT.tiff]

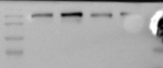

Supplement: Figure 4—source data 1. [file elife-69906-fig4-data1.zip › Figure 4-source data 1/fig4 E/p-EGFR.tiff]

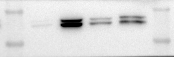

Supplement: Figure 4—source data 1. [file elife-69906-fig4-data1.zip › Figure 4-source data 1/fig4 E/P-ERK.tiff]

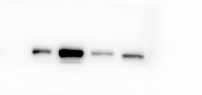

Supplement: Figure 4—source data 1. [file elife-69906-fig4-data1.zip › Figure 4-source data 1/fig4 E/VCAM-1.tiff]

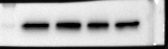

Supplement: Figure 4—source data 1. [file elife-69906-fig4-data1.zip › Figure 4-source data 1/fig4 G/AKT.tiff]

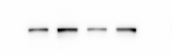

Supplement: Figure 4—source data 1. [file elife-69906-fig4-data1.zip › Figure 4-source data 1/fig4 G/EGFR-10.tiff]

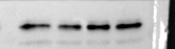

Supplement: Figure 4—source data 1. [file elife-69906-fig4-data1.zip › Figure 4-source data 1/fig4 G/erk.tiff]

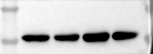

Supplement: Figure 4—source data 1. [file elife-69906-fig4-data1.zip › Figure 4-source data 1/fig4 G/GAP-1.tiff]

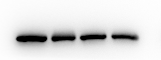

Supplement: Figure 4—source data 1. [file elife-69906-fig4-data1.zip › Figure 4-source data 1/fig4 G/GAP-2.tiff]

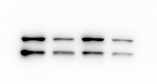

Supplement: Figure 4—source data 1. [file elife-69906-fig4-data1.zip › Figure 4-source data 1/fig4 G/METTL3.tiff]

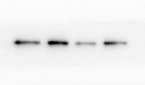

Supplement: Figure 4—source data 1. [file elife-69906-fig4-data1.zip › Figure 4-source data 1/fig4 G/P-AKT.tiff]

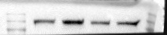

Supplement: Figure 4—source data 1. [file elife-69906-fig4-data1.zip › Figure 4-source data 1/fig4 G/p-EGFR-5-7.tiff]

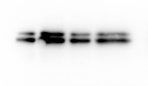

Supplement: Figure 4—source data 1. [file elife-69906-fig4-data1.zip › Figure 4-source data 1/fig4 G/P-ERK.tiff]

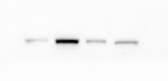

Supplement: Figure 4—source data 1. [file elife-69906-fig4-data1.zip › Figure 4-source data 1/fig4 G/VCAM-1.tiff]

## Slide 1
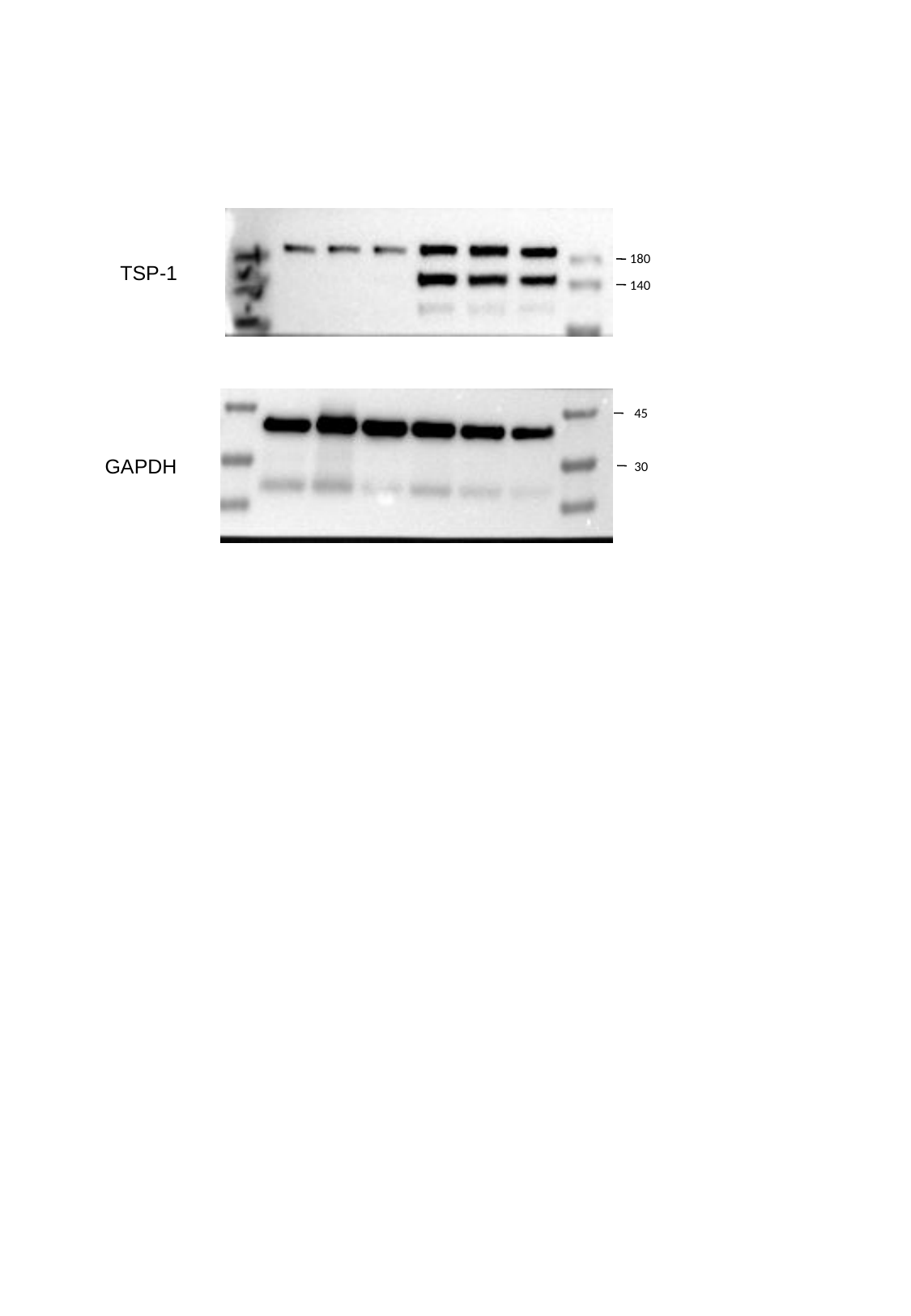

180
TSP-1
140
45
GAPDH
30

Supplement: Figure 4—figure supplement 1—source data 1. [file elife-69906-fig4-figsupp1-data1.zip › Figure 4-figure supplement 1-source data 1.pptx]

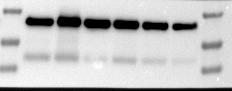

Supplement: Figure 4—figure supplement 1—source data 1. [file elife-69906-fig4-figsupp1-data1.zip › Figure 4-figure supplement 1-source data 1/GADPH.tiff]

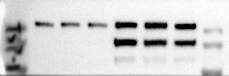

Supplement: Figure 4—figure supplement 1—source data 1. [file elife-69906-fig4-figsupp1-data1.zip › Figure 4-figure supplement 1-source data 1/TSP-1.tiff]
