# Supplementary material for: RNA N6-methyladenosine modulates endothelial atherogenic responses to disturbed flow in mice
Source: eLife. 2022 Jan 10;11:e69906. doi: 10.7554/eLife.69906 (PMC8794471; doi:10.7554/eLife.69906)
Supplement: Figure 4—source data 1. [file elife-69906-fig4-data1.zip › Figure 4-source data 1.pptx]

## Slide 1
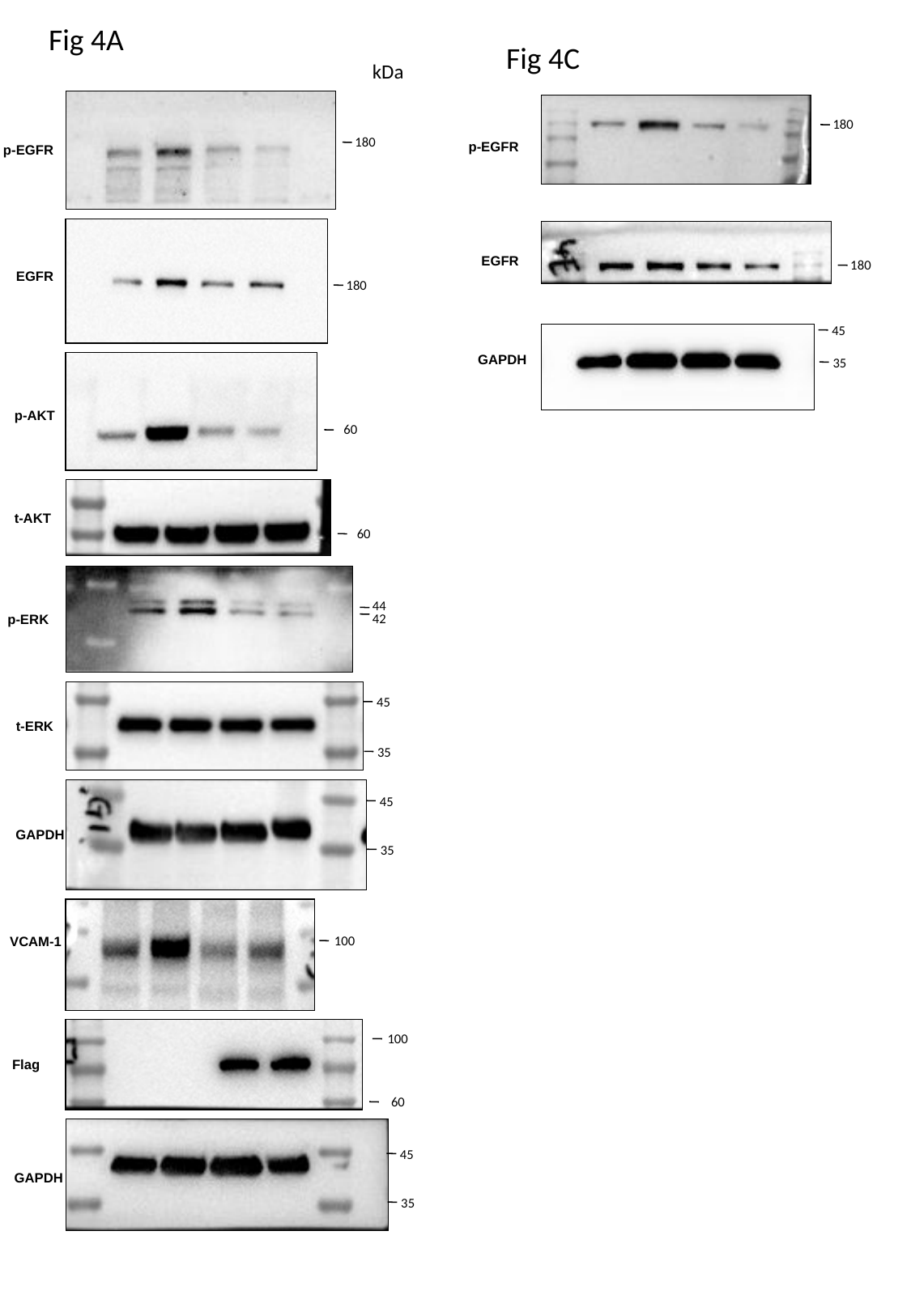

Fig 4A
Fig 4C
kDa
180
p-EGFR
180
p-EGFR
EGFR
180
EGFR
180
45
GAPDH
35
p-AKT
60
t-AKT
60
44
p-ERK
42
45
t-ERK
35
45
GAPDH
35
VCAM-1
100
100
Flag
60
45
GAPDH
35

## Slide 2
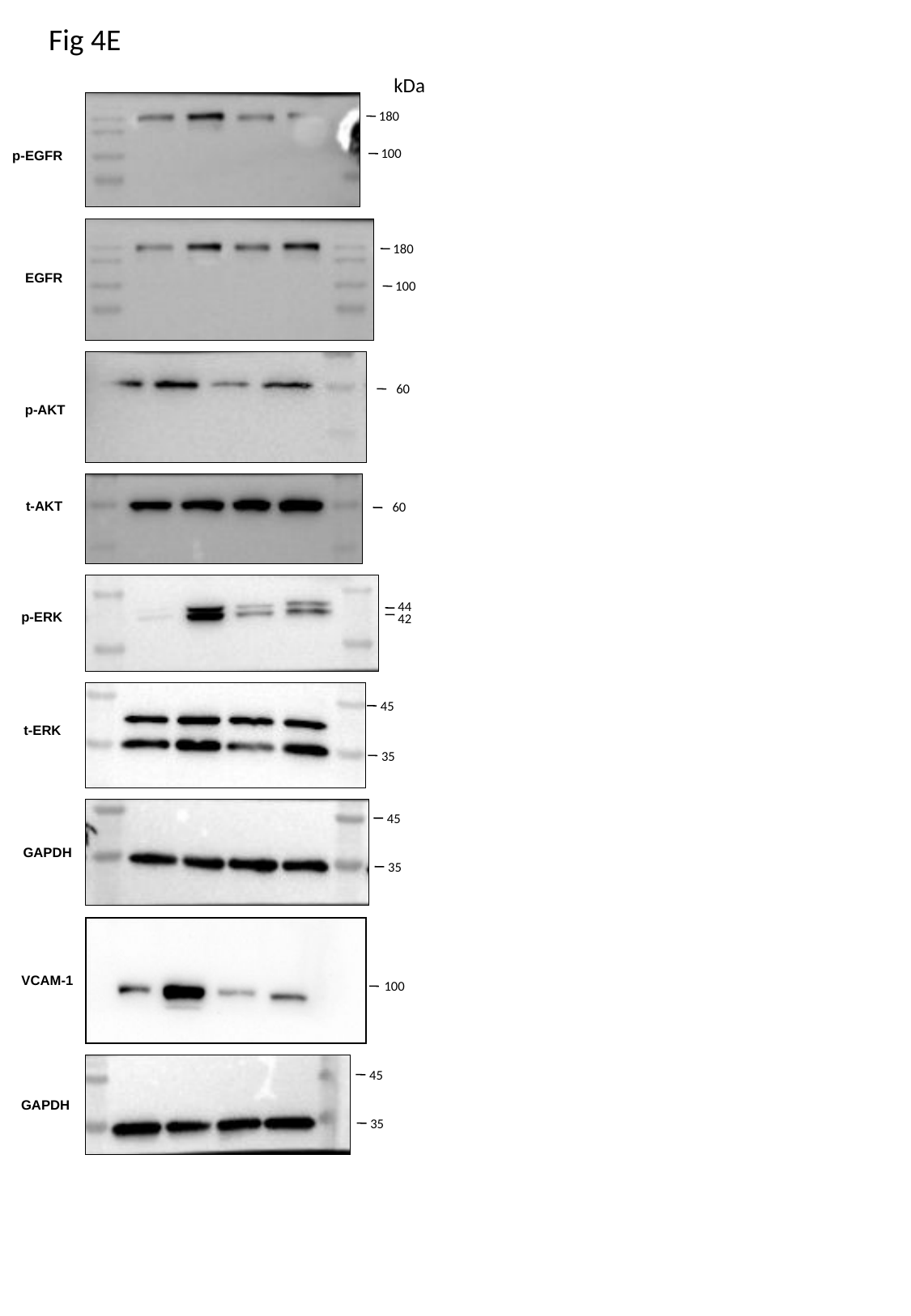

Fig 4E
kDa
180
p-EGFR
100
180
EGFR
100
60
p-AKT
t-AKT
60
44
p-ERK
42
45
t-ERK
35
45
GAPDH
35
VCAM-1
100
45
GAPDH
35

## Slide 3
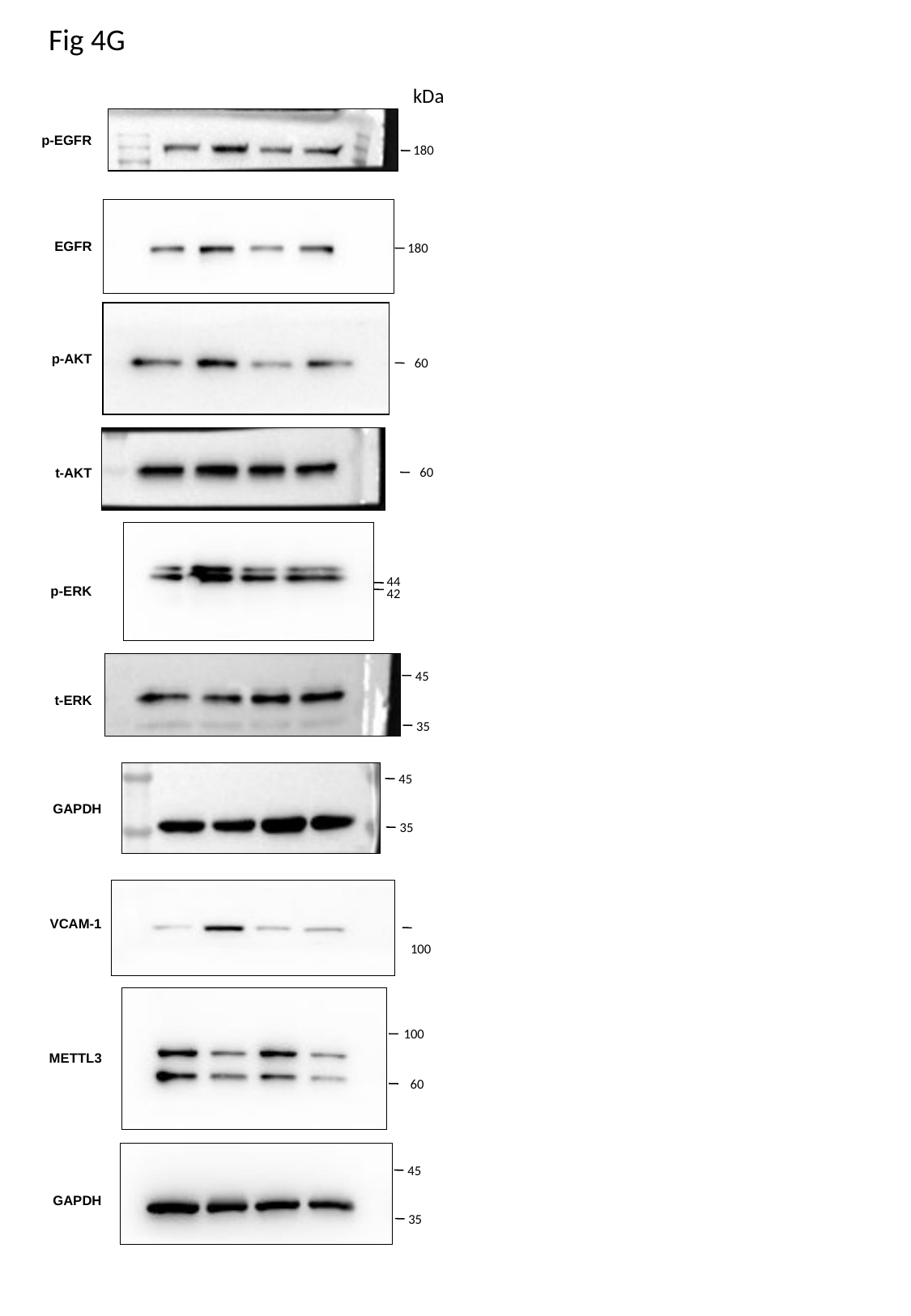

Fig 4G
kDa
p-EGFR
180
EGFR
180
p-AKT
60
t-AKT
60
44
p-ERK
42
45
t-ERK
35
45
GAPDH
35
VCAM-1
100
100
METTL3
60
45
GAPDH
35
